# The rediscovery and delimitation of *Elatostemasetulosum* W.T.Wang (Urticaceae)

**DOI:** 10.3897/phytokeys.126.35707

**Published:** 2019-07-12

**Authors:** Long-Fei Fu, Alexandre K. Monro, Fang Wen, Zi-Bing Xin, Yi-Gang Wei, Zhi-Xiang Zhang

**Affiliations:** 1 Laboratory of Systematic Evolution and Biogeography of Woody Plants, College of Nature Conservation, Beijing Forestry University, Beijing 100083, China Beijing Forestry University Beijing China; 2 Guangxi Key Laboratory of Plant Conservation and Restoration Ecology in Karst Terrain, Guangxi Institute of Botany, Guangxi Zhuang Autonomous Region and Chinese Academy of Sciences, Guilin 541006, China Guangxi Institute of Botany, Guangxi Zhuang Autonomous Region and Chinese Academy of Sciences Guilin China; 3 Herbarium, Royal Botanic Gardens, Kew TW9 3AB, UK Herbarium, Royal Botanic Gardens Kew United Kingdom

**Keywords:** Taxonomy, synonymy, *
Elatostema huanjiangense
*, *
Elatostema tetracephalum
*, China, Guangxi, Guizhou, Rosales, karst landscapes

## Abstract

Of the 280 species of *Elatostema* documented in China, 189 are known only from a single collection. *Elatostemasetulosum* is one such species, having been known only from the type collection for nearly half a century, until recent field investigations in Guangxi. Due to its morphological similarity to *E.huanjiangense* and *E.tetracephalum*, we undertook a critical review of all three species using morphological and molecular evidence. Our results suggest that all three names refer to the same species, which based on priority should be known as *Elatostemasetulosum.* We recognize *E.huanjiangense* and *E.tetracephalum* as synonyms. A distribution map of *E.setulosum* and the extinction risk according to the IUCN criteria is provided. After recircumscription, the taxon must be considered as Least Concern (LC).

## Introduction

*Elatostema* J.R.Forst. & G.Forst. (Urticaceae) is one of two species-rich genera in the Urticaceae, comprising several hundred species of herbs and subshrubs that grow under shade in forests, gorges, stream-sides and caves (Wang 2014, Monro et al. 2018). *Elatostema* is distributed in tropical and subtropical Africa, Australia, Asia and Oceania, but is absent from the Neotropics. Recent phylogenetic research demonstrates that *Elatostema* is a monophyletic group that includes most species of *Pellionia* and excludes *Elatostematoides* and *Procris* (Tseng et al. 2019).

The first revision of Chinese *Elatostema* was undertaken by Wang (1980), at which time 95 species were recognized. This was followed by a second revision in 1995 (Wang and Chen 1995) for Flora Reipublicae Popularis Sinicae, and a third in 2003 (Lin et al. 2003) for the Flora of China, which recognized 137 and 146 Chinese species, respectively. Since then, many new species have been collected and described. Wang (2014) recognized 280 species in the fourth revision of Chinese *Elatostema*. All revisions of the genus indicate southwestern China as the center of Chinese *Elatostema* diversity. This is likely because of its widespread karst landscape, with which 184 out of the 280 species are associated (Wang 2014), and its more tropical climate.

Karst landscapes are characterized by exposed rocks with shallow soils deficient in N and P, but with excessive Ca and Mg that are subject to seasonal droughts and an absence of surface water (Hao et al. 2015, Fu et al. 2017a) in which weathered material is exported subterraneously in solution (Bystriakova N. from The Natural History Museum, London, London, United Kingdom, personal communication), and soil is generated at a very slow rate (Pérez-García and Meave 2005). It is also rich in caves, whose cavern entrances are significant sources of *Elatostema* species diversity and discovery (Monro et al. 2018). Considering the importance of karsts to species discovery and the high frequency of point-endemics amongst karst species (Kong et al. 2017), it is important that this flora is well documented in order for the species conservation to be prioritized effectively and endangered species recognized (Fu et al. 2019a).

Collecting in karst, however, is difficult as there are relatively few roads and the terrain is steeply dissected, the very sharp eroded surfaces making it difficult and dangerous to traverse. As a consequence, there are relatively few collections from such areas and undescribed species are frequently known by only one or two collections. Based on Wang (2014), we find that 2/3 (67%) of species are known from a single collection, and 42% from a single specimen (holotype). Describing a species based on a single collection is problematic as there is no estimate of variation within the species and so there is a risk of applying too many names to the biota (Wei et al. 2011). The over-application of names can make it hard to communicate information about a taxon and to identify specimens. The over-application of names also results in high rates of synonymy that can make taxonomic revision challenging. This is compounded where many taxa in a genus are described from a single collection, as comparisons between taxa become, in effect, comparisons between individual herbarium specimens. Molecular data, however, can provide a means to use paraphyly to identify potentially conspecific groupings (Gao et al. 2012) and to evaluate the phylogenetic informativeness of morphological characters (Scotland et al. 2003).

*Elatostemasetulosum* W.T.Wang was described from a single specimen (holotype) in 1982. This specimen was first identified as ElatostemasessileJ.R.Forst. & G.Forst.var.polycephalum Wedd. in 1964, but later raised to specific rank by Wang in 1982. No additional material was collected until June 2018, at which time a population was discovered close to the type locality. In identifying this recently collected material, we observed that it was morphologically very similar to several species, *E.pergameneum* W.T.Wang, *E.huanjiangense* W.T.Wang & Y.G.Wei and *E.tetracephalum* W.T.Wang, Y.G.Wei & F.Wen, the latter considered to be a synonym of *E.huanjiangense* by Wang (2014). Furthermore, our ongoing research into Chinese *Elatostema* also discovered two new populations of *E.huanjiangense* in Guizhou between 2014 and 2017.

## Material and methods

In order to clarify the relationship among *E.huanjiangense*, *E.setulosum* and *E.tetracephalum*, we undertook a critical examination and comparison of all collections of these related species based on morphological and molecular evidence.

### Sample collection

Fieldtrips in Guangxi and Guizhou were undertaken between 2007 and 2018 to collect specimens of *Elatostemahuanjiangense*, *E.pergameneum*, *E.setulosum* and *E.tetracephalum* which were deposited at BM, IBK, K and PE. For all collections, samples of leaf material were dried in the field using silica gel for use in DNA extraction (Chase and Hills 1991).

### Genomic DNA extraction, PCR amplification and sequencing

Two universal barcodes: the nuclear ribosomal internal transcribed spacer (ITS) region and the *trnH-psbA* intergenic spacer were used to establish hypotheses of evolutionary relationships due to their ability to detect variation at the species level (China Plant BOL Group 2011, Gao et al. 2012). The primers used to amplify the ITS region were those of the China Plant BOL Group (2011). The primers used to amplify the *trnH*-*psbA* intergenic spacer were those developed by Kress et al. (2005). Genomic DNA extraction, PCR amplification and sequencing followed Gao et al. (2012) and Tseng et al. (2019).

### Taxon sampling

To elucidate phylogenetic relationships between the ingroup taxa, *Elatostemahuanjiangense*, *E.pergameneum*, *E.setulosum* and *E.tetracephalum*, we analyzed three accessions of *E.huanjiangense*, and one of *E.pergameneum*, *E.setulosum* and *E.tetracephalum*. As outgroups, we selected *E.grijsii* (Hance) Y.H.Tseng & A.K.Monro and *E.scabrum*(Benth.) Hallier f. based on the most recent published phylogeny for *Elatostema*, *Elatostematoides* and *Procris* (Tseng et al. 2019). Genbank accession numbers for ITS and *trnH*-*psbA*, and voucher specimens information, are listed in Table 1.

**Table 1.** Species name, voucher specimen and accession numbers of *trnH*-*psbA* and ITS used in this study (*denoted newly generated sequences).

Speices name	Voucher specimen	*trnH*-*psbA*	ITS
*Elatostemagrijsii* (Hance) Y.H.Tseng & A.K.Monro	Y.H. Tseng 1167	KC420504	KC420491
*Elatostemahuanjiangense* W.T.Wang & Y.G.Wei	Y.G. Wei g124	KP858730	KP858875
*Elatostemahuanjiangense* W.T.Wang & Y.G.Wei	A.K. Monro & L.F. Fu 7705	MK656519*	MK651815*
*Elatostemahuanjiangense* W.T.Wang & Y.G.Wei	A.K. Monro & L.F. Fu 7719	MK656518*	MK651816*
*Elatostemapergameneum* W.T.Wang	Y.G.Wei 07298	MK656516*	MK651817*
*Elatostemascabrum* (Benth.) Hallier f.	Y.H. Tseng 1219	KC420503	KC420492
*Elatostemasetulosum* W.T.Wang	L.F. Fu et al. FLF180606-01	MK656515*	MK651813*
*Elatostematetracephalum* W.T.Wang, Y.G.Wei & F.Wen	A.K. Monro & L.F. Fu 7696	MK656517*	MK651814*

### Phylogenetic analysis

Sequence data were edited and assembled using the software Lasergene Navigator (DNAStar, Madison, Wisconsin, USA). Edited sequences were then aligned with the MEGA 5.1 (Tamura et al. 2011). The incongruence length difference (ILD) test was implemented in PAUP* 4.0b10 (Swofford 2002) to assess potential incongruence between ITS and *trnH*-*psbA*. The p-value (p = 1) suggested no significant incongruences between datasets. Therefore, we reconstructed the phylogenetic trees based on combined datasets. Phylogenetic analyses were performed using maximum parsimony (MP) and Bayesian inference (BI). MP analysis implemented in PAUP* 4.0b10 which followed Fu et al. (2017b). For BI analyses, the best-fit DNA substitution model HKY+I was selected in Modeltest v 2.7 (Posada and Crandall 1998) according to the Akaike Information Criterion (AIC). BI analyses were conducted in MrBayes 3.2.6 (Huelsenbeck and Ronquist 2001) which followed Wu et al. (2013).

### Distribution map

Distribution map of *Elatostemahuanjiangense*, *E.setulosum* and *E.tetracephalum* was made using the software ArcGIS 10.2 (ESRI, Inc.).

### Morphology examination and conservation assessments

A morphological species concept was employed to compare the taxa based on Wei et al. (2011). Specimens were examined using dissecting microscopy followed Fu et al. (2014, 2017c, 2019b). Extinction threat assessments were undertaken using IUCN criteria (IUCN 2012).

## Results and discussion

### Molecular analysis

**Figure 1.**
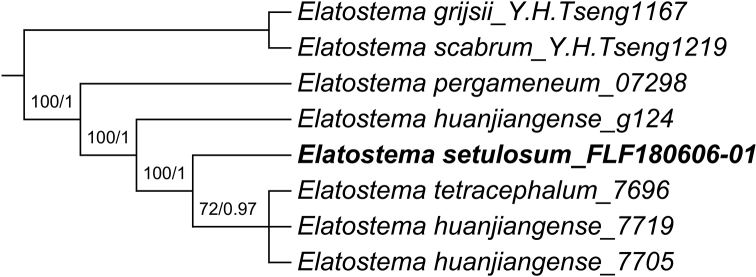
Maximum parsimony phylogenetic tree based on the combined *trnH*-*psbA* and ITS data, showing relationships of *Elatostemahuanjiangense*, *E.pergameneum*, *E.setulosum* and *E.tetracephalum*. Numbers on the branches indicate bootstrap values (≥60%) of the maximum parsimony analysis and the posterior probability (≥0.8) of Bayesian inference analysis.

**Figure 2.**
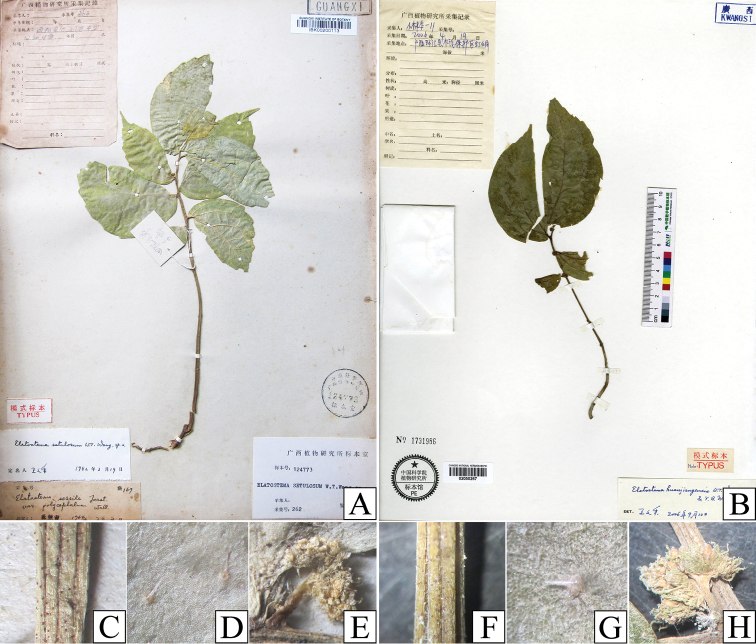
Comparison of type specimens between *Elatostemasetulosum* (**A, C–E**) and *E.huanjiangense* (**B, F–H**): **A, B** habit **C, F** stem **D, G** leaf pubescence **E, H** pistillate inflorescence.

The combined matrix had a length of 1036 characters, 715 for ITS and 321 for *trnH*-*psbA*. Of the 208 (20.1%) variable characters, 117 (11.3%) were parsimoniously informative, including the indels. The maximum parsimony analysis on the combined matrix resulted in three equally parsimonious trees of 241 steps long, a consistency index (CI) of 0.959, retention index (RI) of 0.938 and homoplasy index (HI) of 0.041. MP and BI analyses have same topology (Fig. 1) showing the phylogenetic relationships between *Elatostemahuanjiangense*, *E.pergameneum*, *E.setulosum* and *E.tetracephalum*. The result suggests *E.pergameneum* as sister to remaining ingroup taxa, from which it can readily be distinguished morphologically by its leaves narrower (width less than 30 mm) and adaxial surface glabrous (Wang 1982). Secondly, *E.setulosum* and *E.tetracephalum* are nested within a strongly supported clade that includes a paraphyletic *E.huanjiangense*. After consulting the original descriptions and the type specimens of all three species (Wang 1982, Wang and Wei 2007, Wang 2012), we agreed with the decision of Wang (2014) to consider *E.tetracephalum* as conspecific to *E.huanjiangense*. We were also unable to trace any obvious morphological differences between *E.huanjiangense* and *E.setulosum*, with the exception of leaf pubescence (strigose vs. setulose). Microscope images (Fig. 2) clearly show that the type specimens of both species share the same setulose trichome type. Based on the above, we believe that *E.huanjiangense* and *E.setulosum* represent the same species.

### Taxonomic treatment

#### Elatostema setulosum

Taxon classificationPlantaeRosalesUrticaceae

W.T.Wang, 1982: 120

Figs 2 , 3

##### Type.

CHINA. Guangxi: Tianyang County, Anning Gongshe, *Anon. 262* (holotype: IBK![IBK00200113]). = *Elatostemahuanjiangense* W.T.Wang & Y.G.Wei, 2007: 816. Syn. nov. Type: China. Guangxi: Huanjiang County, Mulun, Hongdong, 19 April 2006, *Y.G. Wei 06128* (holotype: PE![02050267]). = *Elatostematetracephalum* W.T.Wang, Y.G.Wei & F.Wen, 2012: 1100. Syn. nov. Type: China. Guizhou: Huangping County, in forest of earth mount, 20 March 2010, *Y.G. Wei & F. Wen 1067* (holotype: PE!, isotype: IBK!).

##### Description.

Perennial herb, terrestrial, dioecious. Stem 50–250 × 2–3 mm, ascending or erect, simple or branched, glabrous. Stipule 2, persistent, 2–2.2 × 0.2 mm, lanceolate-linear, glabrous. Leaves distichous, alternate, sessile or short petiole; laminae 30–150 × 14–60 mm, length:width ratio 2.1–2.5:1, obliquely elliptic, papery; triplinerve or rarely semitriplinerve; abaxial surface glabrous, adaxial surface sparsely setulose; cystoliths densely scattered, bacilliform; base asymmetrical, broader-half rounded or auriculate, narrower-half cuneate; margin crenate; apex shortly acuminate or acuminate. Staminate and pistillate inflorescences not borne on the same stems. Staminate inflorescences paired, axillary, cymiferous, bearing ca. 30 flowers, ca. 10 mm in diam., peduncle 1.6 × 0.4 mm, subglabrous; bracts membranous, linear-lanceolate or lanceolate-linear, 1.2–2.5 × 0.3–1 mm, sparsely ciliate; staminate flower bud ovoid, flowers ca. 1.6 × 1 mm, glabrous, tepals 5, subapical appendage ca. 1 mm, corniculate. Pistillate inflorescences paired, axillary, capitate, bearing ca. 20 flowers, inflorescences with three types: (1) simple capitulum, 1.5 mm in diam., receptacle inconspicuous, bracts ca. 10; (2) composite capitulum, comprised by four 2-branched simple capitulum; (3) simple capitulum, receptacle discoid-oblong, 2–3 × 0.8–2 mm, weakly divided into two lobes, glabrous, subtended by marginal bracts; bracts numerous, triangular, ca. 0.5 × 0.2–0.3 mm, glabrous; bracteoles 2 per flower, subequal, 0.5–1 mm, linear, semitransparent; Pistillate flowers: ovary ovoid, ca. 0.6 mm; achene 6.122–7.99 × 3.891–5.119 mm, length:width ratio 1.56–1.57:1, broadly ellipsoid, with 4 longitudinal ribs and tuberculate, two opposite longitudinal ribs winged.

##### Additional specimen examined.

CHINA. Guangxi: Huanjiang County, Mulun nature reserve, Hongdong, 8 April 2009, *Y.G. Wei g124* (IBK!, PE!); Huanjiang County, Mulun nature reserve, Leyi Village, Donglai, 3 May 2011, *Y.S. Huang*, *Y.B. Liao & R.C. Peng y0216* (IBK!); Huanjiang County, Mulun nature reserve, Leyi Village, Donglai, 18 April 2012, *W.B. Xu*, *R.C. Peng & R.C. Hu ML1037* (IBK!); Huanjiang County, Mulun nature reserve, Hongdong, 16 April 2012, *L.F. Fu FL004* (IBK!, PE!); Huanjiang County, Chuanshan Town, on the way from Hongdong to Zhonglun, 9 May 2006, *Man-Fu Hou 117* (PE!); Tianyang County, Babie Village, Anning Village, 6 June 2018, *L.F. Fu*, *Y.C. Liu & W.J. Xu FLF180606-01* (IBK!); Guizhou: Huangping County, Feiyun gorge, 17 May 2012, *Y.H. Tseng & L.F. Fu Zn1217* (IBK!), 8 Nov. 2015, *A.K. Monro & L.F. Fu 7696* (IBK!, K!); Shibing County, Yun Tai Mountain, 10 Nov. 2015, *A.K. Monro & L.F. Fu 7705* (IBK!, K!); Zhen Yuan County, Tiexi Tourist Park, 10 Nov. 2015, *A.K. Monro & L.F. Fu 7719* (IBK!, K!).

##### Conservation status.

**Figure 3.**
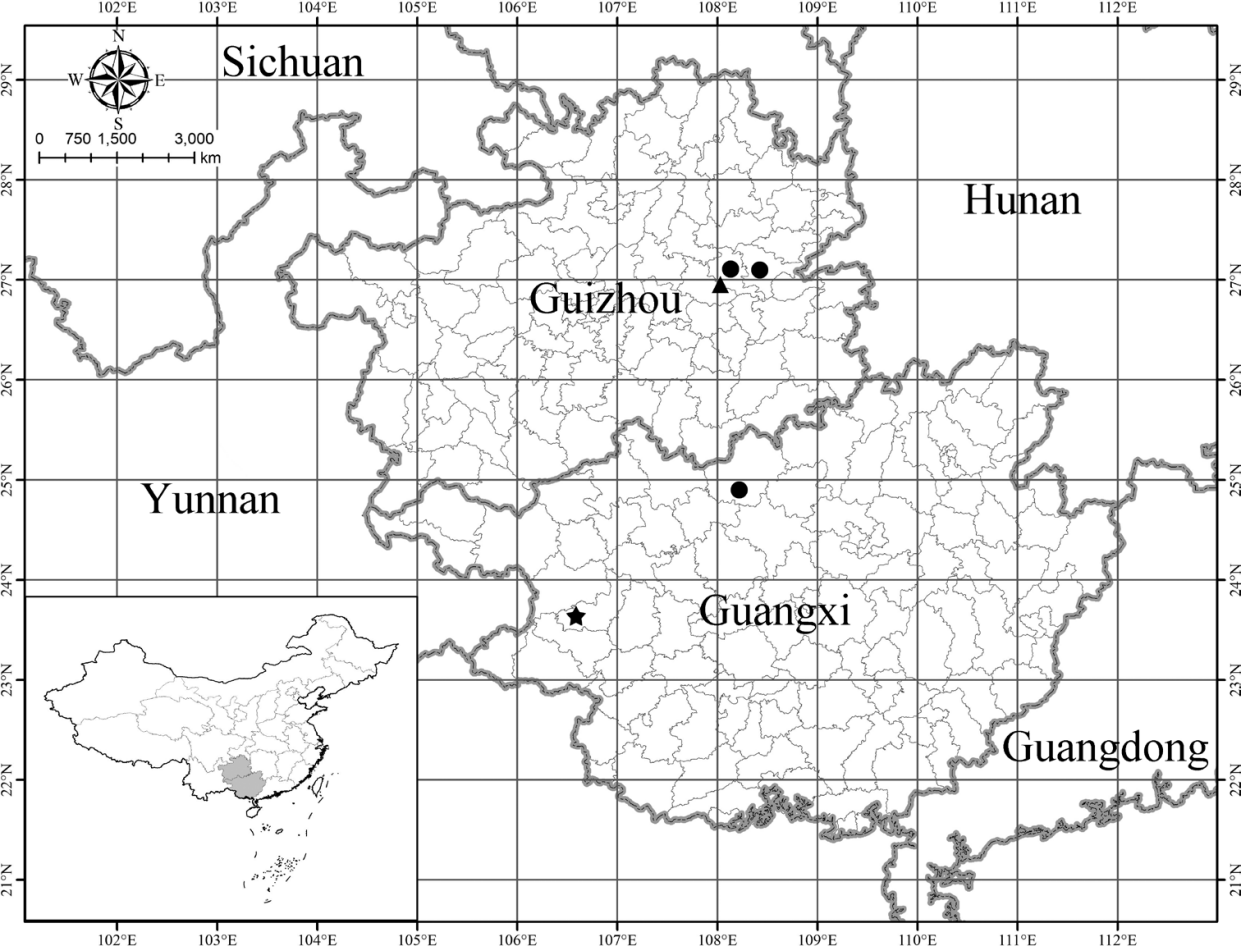
Distribution map of *Elatostemahuanjiangense* (circle), *E.setulosum* (star) and *E.tetracephalum* (triangle).

An Extinction Threat Assessment was undertaken using the IUCN methodology (2012). *Elatostemasetulosum* is known from five localities in Guangxi (one population) and Guizhou (four populations), China. We estimate that the population of mature individuals is greater than 1000. The given surface area of a polygon including the known localities for this species is greater than 27,000 km^2^ (Fig. 3); it is also likely that there remain as yet undiscovered populations. For these reasons we assess *E.setulosum* as Least Concern (LC).

## Supplementary Material

XML Treatment for Elatostema setulosum
